# A thematic synthesis of qualitative studies and surveys of the psychological experience of painful endometriosis

**DOI:** 10.1186/s12905-023-02874-3

**Published:** 2024-01-18

**Authors:** Amanda C. de C Williams, Honor McGrigor

**Affiliations:** Research Dept of Clinical, Educational & Health Psychology, University College London, Gower St, London, WC1E 6BT UK

**Keywords:** Pelvic pain, Infertility, Social impact, Quality of life, Delayed diagnosis, Treatment failure, Fear of disease progression

## Abstract

**Background:**

Endometriosis is a widespread problem in women of reproductive age, causing cyclical and non-cyclical pain in the pelvis and elsewhere, and associated with fatigue, fertility problems, and other symptoms. As a chronic pain problem, psychological variables are important in adjustment and quality of life, but have not been systematically studied.

**Methods:**

A systematic search of multiple databases was conducted to obtain surveys and qualitative studies of women’s experience of pain from endometriosis. Surveys were combined narratively; qualitative studies were combined by thematic synthesis, and the latter rated for methodological quality.

**Results:**

Over 2000 records were screened on title and abstract, and provided 22 surveys and 33 qualitative studies from which accounts could be extracted of the psychological components of pain in endometriosis. Surveys mostly addressed quality of life in endometriosis, with poorer quality of life associated with higher levels of pain and of distress, but few referred to coherent psychological models. Qualitative studies focused rather on women’s experience of living with endometriosis, including trajectories of diagnosis and treatment, with a few addressing meaning and identity. Thematic synthesis provided 10 themes, under the groupings of internal experience of endometriosis (impact on body, emotions, and life); interface with the external world (through self-regulation and social regulation); effects on interpersonal and social life, and encounters with medical care.

**Conclusions:**

The psychological components of pain from endometriosis only partly corresponded with standard psychological models of pain, derived from musculoskeletal pain studies, with fewer fears about physical integrity and more about difficulties of managing pain and other symptoms in social settings, including work. Better understanding of the particular psychological threats of endometriosis, and integration of this understanding into medical care with opportunities for psychologically-based pain management, would substantially improve the experience and quality of life of women with painful endometriosis.

**Supplementary Information:**

The online version contains supplementary material available at 10.1186/s12905-023-02874-3.

## Background

Endometriosis affects about 5–8% women of reproductive age [1–4]. Typical symptoms include dysmenorrhea, noncyclic pelvic pain, dyspareunia, fatigue, pain on emptying the bladder or bowels, and heavy bleeding [5, 6]. Pain is not only felt in the pelvis, but can be localized to various other parts of the body, such as the lower back. Pain and other symptoms can be felt constantly, cyclically with worsening around menstruation and/or ovulation [6], or unpredictably, and they vary between and within people with endometriosis. Additionally, endometriosis can be comorbid with other chronic (persistent) pain conditions, including tension headache and migraine, fibromyalgia, myofascial pain, vulvodynia, bladder pain, and low back pain, often referred to as Chronic Overlapping Pain Conditions (COPCs) and attributed to changes in central sensitization [3, 7, 8].

Diagnosis is commonly delayed for several years after symptom onset, and access to imaging or surgical diagnosis may be restricted by social inequalities [3] as well as by limited knowledge of endometriosis in primary care, with difficulty both for women and for healthcare staff distinguishing endometriosis from putatively ‘normal’ dysmenorrhea [1, 9–11]. Following diagnosis, pain reduction sometimes takes second place to treating the condition, but neither size of lesions [12] nor stage of disease predicts frequency, constancy, or intensity of pain. Finally, pain often persists or recurs after effective surgical, hormonal, or other treatments [13].

In any chronic pain scenario, psychological variables are important in determining the personal experience of chronic pain, overall adaptation, and prognosis [14, 15]. Difficulty living with chronic pain may or may not reach clinical criteria for depression or anxiety [16], but tends to be focused on pain and its implications for overall health, including fertility, as well as on difficulties in everyday life and in lifetime goals. Intimate and social relationships are disrupted, as is work, with implications for career and financial security. Women can find it hard to communicate their pain and other symptoms, or to find someone who has the appropriate skill to handle their struggle [17]. Therefore, people with endometriosis may become isolated and distressed [1, 18–20]. Overall, quality of life is reduced, although that is not necessarily routinely reflected in studies of natural history or treatment outcome [21]. Additionally, its association with menstrual bleeding renders it stigmatized [19], while female pelvic pains in general are at risk of being dismissed as mental health problems (22, 17].

Within the broader biopsychosocial framework, the dominant psychological model in chronic pain is that of fear and avoidance [14, 22], whereby overestimation of the threat inherent in pain for physical integrity leads to avoidance of a wide range of activity, which in turn leads both to increasing disability (through deconditioning) and to depression (through losses inherent in avoidance). Both anxiety about pain and damage and losses due to restricted activity contribute to worsening of pain experience (particularly by descending modulation of pain) and to maintaining anxiety and restricted activity. However, this is largely based on studies of musculoskeletal pain, often low back pain, in which people with pain associate that pain with damage to essential joint and vertebral structures, provoking caution around movement; for instance, fewer than 10 of 335 studies of pain-related fear and avoidance concerned visceral pain, and none endometriosis [23]. It is not clear to what extent the same psychological model applies to visceral pains, where fears of damage may be less prominent, and fears of disease and long-term prognosis where disease is diagnosed may be far more salient; nor is it clear what activities are routinely avoided and how that affects everyday life in both short and long term.

In early studies, very similar mood and social adjustment outcomes were found in women with diagnosed endometriosis compared with those with negative laparoscopy for pelvic pain [24], and, despite a high level of concern about undiagnosed disease, few gynecology patients with pain endorsed worry about cancer [25]. No psychological models specific to endometriosis, or to painful gynecological conditions, have developed, and a 2015 systematic review and thematic synthesis of qualitative research in endometriosis [26] noted the lack of studies of emotional and social wellbeing. One review since has provided more information on psychological impact of endometriosis. A narrative synthesis of 16 qualitative studies [1] described themes of powerlessness, and of loneliness and isolation, but not of anxiety about pain, and worry only in relation to infertility. A more recent systematic review included meta-analyses showing higher depression and anxiety scores in women with endometriosis when compared with healthy controls, but not when compared with other women with chronic pelvic pain [16]; the focus of depression and anxiety were not described, although their correlation in at least some included studies with pain levels and fertility problems was noted.

Several mixed methods or combined quantitative and qualitative reviews add a little more detail of psychological problems associated with endometriosis. One, on coping in women with endometriosis [27], reported catastrophic thinking to be associated with more pain, and “passive” coping and avoidance with poorer mental health. This review [27] also sought studies of metacognition in women with endometriosis, but found none. The same authors, in a large mixed method study, reported that worry about pain, rumination and catastrophizing, were all associated with more distress [28], a result consistent with another review of observational studies [4] and a separate meta-analysis on stress and endometriosis [29]. The only review to take a social focus was an account of stigma causing distress; family members, clinicians, and others who believed endometriosis to be no worse than period pain represented women who struggled with endometriosis pain as exaggerating or complaining excessively [19].

Overall, there is little theorising in this area about the nature of distress or about the fit of existing psychological models of pain in women with painful endometriosis. This literature review aims to elaborate the findings on psychological models used in endometriosis pain described in qualitative and survey research, and to outline outstanding areas that need further investigation, using a systematic method of synthesizing the findings of qualitative studies [30].

## Methods

This literature review was pre-registered (PROSPERO CRD42022330527), and in preparation for the review, the researchers discussed endometriosis and key literature with expert clinicians and experts by experience, and consulted an academic librarian about the search terms and databases to use. Reporting of the review is in accordance with the ENTREQ statement [31] (see additional files Table 2).

### Search strategy

On 6th May 2022, a comprehensive literature search of all years of Medline, Embase, PsycInfo, PsycExtra, ProQuest Dissertations & Theses Global, and LILACs was conducted, aiming to include grey literature and international databases. Broad search terms on endometriosis, pelvic pain, quality of life and experience were employed, and as they returned a large number of results, references of studies retrieved were not further searched. The search terms used are shown in additional files Table 1.

### Inclusion and exclusion criteria

The inclusion criteria were qualitative research or surveys, from peer reviewed journals or publicly available PhD theses, whose participants were adult women (18 and over), not solely concerned with healthcare experience. There were no limitations placed on language or date of publication. We excluded studies unrelated to endometriosis, studies of non-human animals, literature reviews, and conference abstracts.

### Study selection

**Fig. 1:**
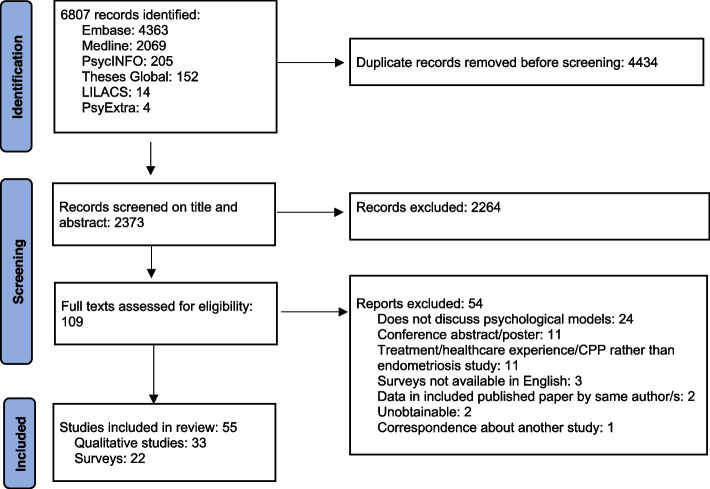
Search and selection of qualitative studies

This search returned a large number of records, initially screened using the Endnote X9.3.3 deduplication function, with further duplications removed by hand. Titles were screened to remove theses below PhD Level and irrelevant literature (e.g. male pelvic pain, cancer etc.). The next stage of study selection (see Fig. 1) involved one researcher (HM) screening titles and abstracts, removing those that were solely treatment comparisons, that focused on chronic pelvic pain (CPP) with no reference to endometriosis, or that focused exclusively on interactions with healthcare professionals. Another researcher (AW) checked a ~ 15% random sample (300) of the rejected titles plus all 270 studies identified as possibly meeting criteria; decisions were discussed and agreed. Full papers were read for all possibly eligible studies, with further removal of conference abstracts, papers with no reference to psychological models, accounts only of treatment or healthcare experience, or chronic pelvic pain without separate description of participants with endometriosis.

### Quality assessment

The surveys and qualitative research were then analysed and synthesized separately. For the qualitative research, an amalgamation of the CASP and COREQ quality assessment tools (Appendix A) was used to assess the quality of the studies. The CASP and COREQ quality assessment tools were both selected as appropriate after trying several other tools on four studies. Duplicate questions on the two tools were removed, the combined version test run on four further studies, nonessential items removed, and then the entire set split for rating, with an overlap of four studies to check consistency. Lower quality studies were not removed, but the rating was kept in mind during analysis. The researchers both assessed four randomly selected studies, then compared findings. Following this, they split the studies, and individually assessed them.

### Surveys: narrative analysis

For the surveys, data were extracted using narrative synthesis methods [32], suitable for thin data, on the population, sample size, location of study, questionnaire and research tools used, the preoccupation/themes of the survey questions and psychological models discussed.

### Qualitative data synthesis

The data was synthesized according to Thomas and Harden’s [30, 33] thematic synthesis method using inductive coding. This was chosen after reading around the topic (particularly [33, 34]) and discussion with a colleague experienced in the fields of pain and of methods of qualitative synthesis.

First, line by line codes were developed by the first researcher (HM), and used on the Results and Discussion sections of all included papers, recording in NVivo 12 1.6.1. The resulting longlist contained 189 codes. The second researcher (AW) applied these codes to 20% of the sample primary studies, suggesting new ones where necessary. The codes were discussed, agreed, and collapsed or combined. Descriptive themes, staying close to the content of the primary studies, were then generated from the grouped codes, separately by each researcher, then discussed and agreed. Finally, analytic (interpretative) themes were developed jointly using the map of descriptive themes and their constituent codes. Themes were, where possible, given a title that used the words of a participant from one of the primary studies.

### Positionality and reflexivity

Given the subjective bias inherent in decisions described above, and of interpretations, we include a statement of position to make our perspectives more transparent. AW is an academic and clinical psychologist, with over 35 years’ experience working in chronic pain, including chronic pelvic pain. While she has used the fear and avoidance model in academic and clinical work, she considers it to capture only part of the chronic pain experience, even in musculoskeletal pain. HM is a research assistant, with experience in qualitative research. This was her first project researching pain, and considers the biopsychosocial model to be the most convincing pain model to date. Throughout the project, the researchers aimed for reflexive processing of reviewed material, considering at each point whether and how their beliefs and concerns might influence their decisions.

## Results

Over 2000 records were screened on title and abstract, and 109 selected as possibly eligible. These were read as full papers. Despite help from libraries, and attempts to contact authors, full texts for seven studies could not be obtained. Two studies were discovered in the search as theses, but authors directed us to their published studies, which were included. Three survey studies, all with abstracts but not full text in English, appeared to be unlikely to meet criteria so were excluded. Responses were not forthcoming from two sets of authors. This resulted in the synthesis of 22 surveys and 33 qualitative texts, one of which was translated from Portuguese.

### Surveys

The research literature that used surveys of women with endometriosis (with or without a comparison population) to elicit information about physical and psychological health were predominantly concerned with quality of life and what physical and psychological variables were associated with it. This appeared to be an area of increasing interest: seven studies were published between 2016 and 2019, six studies each in 2020 and 2021, and three in 2022 up to the point of the search. Six studies were from Australia, four from the USA and Canada, one from Brazil, one from South Africa, and the remainder from Europe. All but one, on adolescents up to 25 years old, recruited adults, usually defined as over 18 years, mainly relying on self-diagnosis, with some self-report of medical diagnosis. All studies were cross-sectional, 12 studies describing a single population, eight comparing women with endometriosis with women without, and two making comparisons within a sample of women with endometriosis, one relating to psychological health and the other to diet.

The most common focus of the studies was quality of life and the gynecological, pain and psychological symptoms associated with it (nine studies), with two investigating sexual activity in relation to quality of life. Pain and its relationship to lifestyle problems in endometriosis was addressed by three studies; psychological problems, depression in particular, were the focus of two studies and stigma of one further study. Fatigue was investigated in two studies; and diet and infertility in one each. One study tested the performance of a generic psychological questionnaire in an endometriosis population.

Pain (pelvic pain, abdominal pain, low back pain, menstrual pain, dyspareunia, and pain on defecation) was investigated in relation to quality of life in 10 studies [34–43], all of which found more pain to be associated with poorer quality of life (often health-related quality of life), greater impact of endometriosis on life, or poorer psychological health. Two of these 11 [35, 39] reported dyspareunia alone to be associated with poorer quality of life. One further study [44] did not analyse pain separately from a broader physical function score which was associated with poorer quality of life. Two other studies on pain [45, 46] investigated characteristics of the pain itself and reported evidence of central sensitization.

The association of endometriosis symptoms with psychological symptomatology was investigated in eight studies [37, 42–44, 47–50], generally finding greater distress (although Bien [34] did not) and an association between greater distress, more or more severe endometriosis symptoms and particularly pain, and poorer quality of life. Only one study [41] was explicit about the psychological model used as a basis for the investigation, describing the fear and avoidance model [51] and using catastrophizing [52] as a central variable. They described phenomena related to the fear and avoidance model in terms of pain cognition: hypervigilance to pain, catastrophizing, and fear of pain, all of which they found to be more extensive in women with endometriosis than in healthy controls; avoidance of activity on the basis of fear of pain was inferred, not sampled. Other papers in this sample drew implicitly or explicitly on psychological distress as a common consequence of endometriosis, but for at least one survey psychological distress was assumed to be an antecedent [42]. (See additional files for surveys not referenced here.)

### Qualitative studies

The largest number of studies (11) was conducted in the UK; Australia and the USA provided 5 each, with 3 from Brazil, 2 each from Italy and Germany, and one each from New Zealand, Iran, Puerto Rico, Spain, The Netherlands, Sweden, Hungary; and one sampled from France, Germany, and the USA. They were published from 1995 to 2022, the majority since 2018, with a range of participants from six to 61, a mean of 25 per study. Participants were mainly recruited through advertisements in endometriosis groups and online message boards and social media (19 studies), with 7 studies using patients identified/referred by medical professionals, 7 recruited from outpatient clinics or hospitals, 5 using word of mouth/snowballing alongside those listed above, 4 using a subset of a larger study, and 2 using a medical recruitment company. For the three studies [53–55] that included healthcare professionals in their sample, we used as far as possible only material from women with endometriosis. Twenty-three of the 33 studies collected data through individual interview, with a mix of face-to-face and online settings; five used focus groups; three used a written response, and two used combinations of these methods. Participants were mainly in their thirties or early forties, with a range of 12–78 years (23 studies provided data) and a mean of 35 years (from 21 studies). The mean age at diagnosis was 27 years (8 studies), with a mean diagnostic delay of 8.5 years (8 studies) and mean age for onset of symptoms 17 years old (2 studies). Ethnic diversity was purposively sampled in just one study [56]; where ethnicity was reported, there was a general lack of diversity, but this was rarely commented on (one exception was Cole et al. [57]) (Table 1).

**Table 1:** Characteristics of qualitative studies

Author	Title	Year	Research focus	Recruitment	Sample size	Data Collection Method
Bento & Moreira [58]	Quando os olhos não veem o que as mulheres sentem: a dor nas narrativas de mulheres com endometriose	2018	Pain	Community - Internet support groups	20	Interview
Boersen et al. [59]	Patients’ perspective on cognitive behavioural therapy after surgical treatment of endometriosis: a qualitative study	2021	CBT	Clinical - from a referral centre for endometriosis -	17, in 5 focus groups	Focus groups
Bullo & Hearne [60]	Parallel worlds and personified pain: A mixed methods analysis of pain metaphor use by women with endometriosis	2021	Language	Community - self-selecting, advert on social media	21	Interview
Clark [61]	Experiences of women with endometriosis: An Interpretative Phenomenological Analysis.	2012	General experience	Community - Endometriosis UK support groups, and adverts in local areas	13	Interview
Cole et al. [57]	“The most lonely condition I can imagine”: Psychosocial impacts of endometriosis on women’s identity	2020	Identity	Community- online - advertised through charity	34	Open written response
Cox et al. [62]	Focus group study of endometriosis: Struggle, loss and the medical merry-go-round	2003	Experiences of endometriosis and laparoscopy	Clinical - patients of specialist unit invited	61 in 5 focus groups	Focus group, interview, survey
Denny [63]	Women’s experience of endometriosis	2004	Living with endometriosis	Community and clinical - online message board, snowball	15	Interview
Di Biasi [64]	The meaning of endometriosis to females experiencing the disease	1995	Living with endometriosis (specifically for nurses)	Community - advertised through charity and support group	33	Open written response
Di-benedetti [65]	Patients’ perspectives of endometriosis related fatigue: qualitative interviews	2020	Fatigue	Clinical - medical recruitment company	22	Interview
Drabble et al. [66]	Constellations of pain: a qualitative study of the complexity of women’s endometriosis-related pain	2021	Complexity of pain experience	Community - support groups, Facebook, snowball	20	Interview
Eastwood [67]	Endometriosis: Medical Delegitimation and the Reconstruction of Narrative Identity	2005	Living with endometriosis – social	Community - advert in newspaper	35	Open written response
Gater et al. [68]	Development and content validation of two new patient-reported outcome measures for endometriosis: the Endometriosis Symptom Diary (ESD) and Endometriosis Impact Scale (EIS)	2020	Development of research tools	Clinical - referrals from treating physicians	45	Interview
Guan et al. [69]	The endometriosis daily diary: qualitative research to explore the patient experience of endometriosis and inform the development of a patient-reported outcome (PRO) for endometriosis-related pain	2022	Development of research tools	Clinical - recruitment agency - referrals from medical professions	30	Interview
Hållstam et al. [70]	Living with painful endometriosis – A struggle for coherence. A qualitative study	2018	Pain	Clinical - pain clinic	13	Interview
Hudson et al. [56]	Endometriosis: improving the wellbeing of couples	2013	Experience of partner/couples	Community and Clinical - support groups, hospital clinics, word of mouth	22	interview
Hunting-don & Gilmour [71]	A life shaped by pain: women and endometriosis	2005	Living with endometriosis (nursing literature)	Community - support group	18	interview
Jaeger et al. [72]	“A little monster inside me that comes out now and again”: endometriosis and pain in Austria	2022	Living with endometriosis, particular focus on pain	Community - outpatient clinic attendees	10	interview
Jones et al. [73]	The impact of endometriosis upon quality of life: a qualitative analysis	2004	Living with endometriosis – impact on quality of life	Clinical - online advert, Facebook, snowball	24	interview
Mander-son et al. [74]	Circuit Breaking: Pathways of Treatment Seeking for Women With Endometriosis in Australia	2008	Diagnosis	Community - from a larger study, also newspapers and noticeboards and snowball	40	interview
Márki et al. [75]	Challenges of and possible solutions for living with endometriosis: a qualitative study	2022	General experience	Clinical - from a larger study	21	Focus groups
Markovic et al. [76]	Endurance and contest: women’s narratives of Endometriosis	2008	Illness narratives	Clinical - from a larger study	30	interview
Matias-Gonzales et al. [77]	“Es que tú eres una changa”: stigma experience	2021	Stigmatisation/taboo	Community - flyers	50 (10–12 per focus groups)	Focus groups
Mellado et al. [78]	Social isolation in women with endometriosis and chronic pelvic pain	2015	Social isolation	Clinical - patients at a clinic	29	Focus groups
Moradi et al. [79]	Impact of endometriosis on women’s lives: a qualitative study	2014	General experience (across different ages)	Clinical and community - endometriosis centre, also information night, & recommendations from a doctor	35	Focus groups
Olliges et al. [80]	The Physical, Psychological, and Social Day-to-Day Experience of Women Living With Endometriosis Compared to Healthy Age-Matched Controls—A Mixed-Methods Stud	2021	Experience across the menstrual cycle	Clinical - outpatient centres	12 endometri-osis patients, 11 age-matched healthy controls	interview
Osborne [81]	The effects of symptomatic endometriosis on womanhood	2008	Identity/womanhood	Clinical through clinic and outpatients - identified by doctor	7 (response rate 50%)	interview
Rea et al. [82]	Living with endometriosis: a phenomenological study	2020	General experience	Clinical - identified by doctor/healthcare providers	25 (data saturation achieved)	interview
Riazi et al. [53]	Patients’ and physicians’ descriptions of occurrence and diagnosis of endometriosis: a qualitative study from Iran	2014	Diagnosis	Clinical - at a hospital	6 gynaeco-logists, 12 patients	interview
Rowe et al. [53]	Improving clinical care for women with endometriosis: qualitative analysis of women’s and health professionals’ views	2021	Compare women’s perceptions of healthcare with health professionals’	Community - advertisements on Facebook	46 women, 13 health profess-ionals	combination of focus group and open written response - questions posted online, recorded, prompts and additional questions
Silva et al. [83]	Experiences of women regarding their pathways to the diagnosis of endometriosis	2021	Diagnosis	Community - recruited online support groups, adverts	10	interview
Varney [84]	Women’s experiences of endometriosis: Qualitative explorations of psychological support, and interactions with healthcare professionals	2020	General experience – with focus on support systems	Clinical - identified by healthcare workers	15 (5 withdrew prior to interviews - reasons given)	interview
Zale et al. [55]	Shedding light on endometriosis: Patient and provider perspectives on a challenging disease	2019	General experience – comparison of healthcare providers and patients	Community - recruitment flyer on social media pages of endo organisation	4 providers, 12 patients	interview
Zarbo et al. [28]	Cognitive and Personality Factors Implicated in Pain Experience in Women With Endometriosis: A mixed method study	2019	Links between experience of pain and psychological/cognitive factors	Clinical - from larger study	6	interview

### Quality assessment

Information collected using the combined COREQ/CASP form is provided in full in Additional files, Table 4, and summarized here. Interviewers identified themselves as academics, students, psychologists or nurses, although about half the studies provided no information, and few described any training in interviewing. Twenty studies (see Additional files Table 4) employed only female interviewers, one used both male and female, and one only male [59]; the remainder did not specify the sex of the interviewer/s. Five researchers identified themselves in their publication as having endometriosis [57, 60, 61, 64, 67], but it is not clear whether that information was shared with their interviewees, making it hard to estimate how it might have affected data. Two studies reflected on whether characteristics of the interviewer/s might have biased recruitment or interview content, one [57] in relation to ethnicity, declaring interviewers to be white academics, and the other [81] in terms of sociodemographic characteristics. Seven further studies included reflexive comments on the researchers, and six more a very limited statement; others provided none, despite the convention of qualitative researchers attempting to be transparent about possible biases brought to their data.

Four studies described their methods only as qualitative; the remainder elaborated, either identifying a method (such as discourse analysis, or thematic analysis) or an epistemological stance, or both. The most common aim was to describe women’s experience of living with endometriosis (14 studies), including its social impact; the next most common was studies of assessment tools or trajectories of diagnosis and treatment (8 studies); there were 7 studies of meaning and identity; two of language use; and one each of stigma and of fatigue.

### Thematic synthesis

**Fig. 2:**
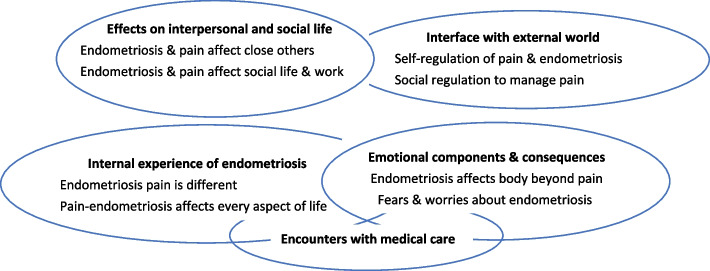
Main themes and sub-themes

Coding of content of results and discussion, both directly reported participant comments and those of the researchers, provided 188 initial codes, which were then grouped and named as far as possible using quoted phrases from the studies. Further grouping produced five themes concerned with the internal experience of having endometriosis; two themes about interface with the external world; two concerned with effects on interpersonal and social life, and one (in three parts) with encounters with medical care (see Fig. 2).

#### Internal experience of endometriosis

##### Pain-endometriosis affects every aspect of life

This theme addressed the impact of both endometriosis symptoms and pain on all areas of life, loss of identity, of freedom, and of imagined future. It shared several codes with *Emotional components and consequences of pain and endometriosis.* There was a minor positive component, although this may have been elicited mainly by researchers’ questions about positive aspects.

##### Endometriosis pain is different

The sense of difference from normal menstrual pain was widely emphasized, perhaps because so many women had historically had their early symptoms dismissed as “just period pain”, and perhaps because interviewers were almost always women who would be expected to have experience of dysmenorrhea. Pain was described as qualitatively and quantitatively different, often in very powerful terms, and again this shared several codes with *Emotional components and consequences of pain and endometriosis*.

##### Endometriosis affects my body beyond pain

This theme particularly concerned unpredictable bleeding, in timing or quantity; effects on the bowel, bladder, appetite, energy and sleep, and comorbidities; and discomfort with sexual activity. Women described a relationship with their bodies that had changed for the worse.

##### Emotional components and consequences of pain and endometriosis

Self-doubt, anxiety, depression, and a general sense of being unable to function adequately were commonly reported, directly linked to the problems associated with endometriosis and pain. Occasionally this was expressed with some positive sense of managing it: “yes, it’s painful and yes, it’s awful, but you can live with it”.

##### Fears and worries about pain and endometriosis

Because of our particular research question, we did not subsume this under the previous theme, although that would have been possible. Fears and worries concerned infertility; worsening and recurrence with or without treatment, including the possibility of cancer developing; and concerns that daughters would also have endometriosis. Codes were mainly shared with *Pain-endometriosis affects every aspect of life* and *Encounters with medical care.*

#### Interface with the external world

##### Self-regulation of pain and endometriosis

This theme concerned the ways in which women managed their endometriosis in order to be able to live a more normal life, from “hiding from the world”, to taking analgesics, planning carefully, and building understanding of their condition. In that sense many contributions expressed some sense of achievement of controlling the impact of endometriosis.

##### Social regulation to manage pain and endometriosis

This theme expressed both the scepticism that others in participants’ lives could understand their difficulties, and also help and support received from others in managing endometriosis and pain.

#### Effects on interpersonal and social life

##### Endometriosis and pain affect close others

There was a strong sense, despite self- and social regulation, that family members were negatively affected by the woman’s endometriosis, and in particular, romantic and sexual partners.

##### Endometriosis and pain affect social life and work

Related to the foregoing theme, and to attempts at social regulation, were many accounts of either avoiding socialising at specific times or in general, and of having to take time off work or struggling to hide symptoms. There were a few accounts of friends and of work colleagues and structures being supportive.

#### Encounters with medical care

This was a large theme, perhaps partly as a function of being the main research focus of several studies. It shared few codes with other themes, and had three sub-themes. The first described the overall sense of being unpleasantly exposed by diagnostic and treatment procedures: “I think they forget that you’re a person”. The second sub-theme portrayed diagnosis, often many years after the onset of symptoms, as a turning point. Although most accounts described negative experiences of the struggle for validation and diagnosis, there was also a positive aspect when this was achieved, as in, “I was devastated but relieved”. The third sub-theme concerned disappointments with treatment options and with limitations and disadvantages to what was offered, from contraceptives to encouragement to have children as soon as possible, whatever the woman’s situation. There were very few positive comments on achieving some control through treatment.

### Psychological dimensions of endometriosis pain

The theme of *Emotional components and consequences of pain and endometriosis* provided very familiar material from other studies of chronic pain, musculoskeletal, visceral and pelvic, or mixed [1, 14, 15, 23, 85, 86]. Since we are interested particularly in how well the psychological experience of endometriosis pain fits the generic fear and avoidance chronic pain model [86], we examine here in more detail the content of the theme *Fears and worries about pain and endometriosis.*

The commonest fears, from almost half the studies, concerned infertility. For younger women, this was anticipation of being unable to conceive or being unable to sustain a pregnancy; for older women, infertility was for many (but not all) a significant loss, or for those who had children, concerns about infertility had interwoven in problematic ways both with their treatment options and with their life planning, and some expressed disappointment that parity had not resolved either pain or endometriosis as they had been led to expect. The next most common fear was of recurrence (12 studies) of endometriosis, of worsening following unsuccessful treatment (11 studies) or without (7 studies), and these were linked to extreme pain (“you’d think you were dying”). This most closely resembled the overly negative predictions and associated distress described in the fear and avoidance model. Two other sources of fear occurred in a handful of studies each: of cancer (the diagnosis having been missed, or developing in future), and of genetic transmission of endometriosis to daughters creating additional responsibilities for their mothers in trying to manage it effectively. Not evident in these studies was women’s fear of damage to their bodies during sexual activity, which unlike most other activities that exacerbate pain (such as digestion, or defecation) can be avoided. Attempts at control for such activities focus rather on the emotional or social aspects.

## Discussion

Taking together the survey findings and the qualitative meta-synthesis, clear associations emerged between endometriosis pain, distress, and reduced quality of life, but not strongly with any definitive psychological formulation of pain and related problems, nor with the predominant sense of threat that contributes to central sensitization [14]. In the only survey study in which an explicit psychological model was used [41], that of fear and avoidance [86] and catastrophic thinking biases [52], support was found for its application, although methodology was somewhat weak (comparison with pain-free population, and avoidance not directly sampled). Several surveys used outdated models of ‘somatization’, somatic expression of psychological distress, that constitute an unsatisfactory model of endometriosis. The qualitative synthesis combined a relatively large number of studies; they showed substantial common ground in the experiences of women with painful endometriosis, across continents, population samples, and research questions.

No previous review has combined women’s perspectives on the experience of endometriosis pain in such an open-ended way. Our findings describe women’s sense of being let down by and alienated from their bodies, at the same time as needing to attend to and attempt to regulate, or at least predict, their bodies’ vagaries, to function in the outside world, on a daily level, and on a level of life plans. This uses normal rather than psychopathological terms in a coherent framework that combines findings of various other reviews, and is entirely compatible with central sensitization maintaining pain whatever the level of disease [3].

A narrative synthesis of qualitative, quantitative, and mixed method studies [1] described concerns of women with endometriosis about fertility and planning and having children, medical management, information and support, emotional distress (although without any description of anxieties), and feelings of powerlessness. A more recent systematic review [27] of nine quantitative and qualitative studies reported few differences between women with endometriosis pain and people with other chronic pains in metacognitions, including ‘catastrophic thinking’, and coping strategies, noting that more emotion-focused coping and avoidance was associated with poorer mental health. Similar findings are reported in a recent review that distinguished ‘catastrophic thinking’ as the main predictor of pain intensity from anxiety, depression and stress, associated with poorer quality of life [4]. A thematic synthesis by Young et al. [26] noted the gaps in the study of emotional and social wellbeing, and recent studies go some way towards filling this gap [19]. Many reviews of endometriosis note its deleterious effects on quality of life (e.g. [87]), and a few link this directly to pain [16] and, therefore, to the need for psychological support or treatment [88–90].

### Limitations & strengths

Our search was broad and not limited to English language papers, although to those abstracted in English. Nevertheless, there are likely to be studies of endometriosis and associated problems inaccessible to our searches, narrowing the cultural range of studies, and we did not screen references of eligible studies for any missed by the search strategy. Two papers focused predominantly on fertility problems, from Iran and Brazil [53, 83]. We focused only on the experience of women; there is a substantial research literature on the experience of their sexual partners which we excluded. We annotated the studies using a previously untried combination of two established (and somewhat overlapping) scales; this may have increased the arbitrariness of what is taken as a marker of ‘quality’, and contributed to our decision not to assign scores to annotations. We were interested particularly in the extent to which researchers intentionally or unintentionally elicited particularly content in interviews, but reporting of most studies, even the small minority with a reflexive statement, did not comment on this except in the case of a male interviewer [59, 78]. It is hard to summarize quality other than with the narrative provided. We did not double-code and double-rate studies, relying instead on doing so for a sample and proceeding with frequent discussion and consensus, but ideally a larger team would have worked on this review [91]. Finally, the survey data were hard to interpret given that many respondents were self-diagnosed; we have therefore commented more on survey authors’ models than on their outcomes, nor did we attempt any quantitative analyses.

#### Clinical and research implications

Many studies recommended better education about endometriosis for clinicians, emphasizing, in particular, the role of nurses in providing information to patients. We would hope that such education fully integrated the problem of pain and involved all relevant healthcare professionals; our search returned many qualitative studies of women’s experience with endometriosis where pelvic pain was barely addressed, or was represented as one symptom among many, disregarding the extent to which pain itself is a significant cause of distress and difficulty managing everyday life (see [12, 29]), requiring attention and efforts to mitigate pain in its own right, not just secondarily to treatment of endometriosis.

There is a broader need for psychological care to be better integrated into health services for many diagnosable conditions, including endometriosis. Although we found some common ground for understanding psychological problems that can be extrapolated from chronic pain in general, the focus on fear of physical damage and avoidance of physical demands predominant in some psychological interventions is not supported by our findings. Although it is too early in these explorations to suggest specific psychological interventions, information and support, not least from other women with endometriosis and resources created and maintained by them, may meet most needs, with skilled psychological intervention for those women who are more distressed and limited by their pain. Discussion with a clinical specialist about attention to symptoms and when to seek expert healthcare can support self-management in women concerned about recurrence of adhesions; discussion about possible triggers and systematic ways to test them can be helpful to those seeking greater control. Multimodal pain management interventions based in psychological understanding are widely recommended for chronic pain of all sorts (e.g. NICE [92]), but ideally is personalized to the particular problems and priorities of the patient and recognition of specific concerns associated with the disease or type of pain [3].

Several research gaps were noted by As-Sanie et al. [93] at a US meeting of clinicians of various disciplines, women with endometriosis, researchers, and members from industry and government. Among them were the need for mental health professionals attached to endometriosis clinics; the contribution of physiotherapists and others to pain-relieving strategies; the lack of accurate information on relief from different types of hysterectomy, and on pain recurrence following surgery. A priority setting partnership in the UK included in the top 10 priorities, alongside better and less invasive diagnosis and improved education of healthcare professionals, the need to determine the most effective ways of managing the emotional and psychological impact of living with endometriosis [94]. Both effective management and the more routine involvement of mental health professionals proposed by As-Sanie and colleagues [93] require a better understanding of the content of women’s distress about endometriosis, and the particular areas of impact; this review provides a step towards that understanding. There seems little need for repetition of descriptive studies of the impact of endometriosis on women’s lives, given the number and breadth we found. We would recommend investigations with clearer theoretical roots in psychology, particularly but not exclusively the psychology of pain, to establish a solid basis for developing effective psychological interventions, with more of a focus than is characteristic of psychological models to issues of social disclosure, difficulties in social situations, and stigma, affecting both work and personal social situations.

## Conclusion

Endometriosis has widespread impact on women: on their relationship with their bodies; their psychological and social wellbeing; and on life plans and lifestyle. This is similar to the situation of people with other chronic pains, musculoskeletal, neuropathic, or visceral. However, the dominant psychological model of pain, of fear of reinjury and increased pain from avoidable activity, resulting in disability, only partly fits the situation of women with endometriosis. Many factors that exacerbate pain cannot be avoided, nor is there evidence of an overarching fear of physical demands (of everyday life or valued activities) threatening bodily integrity. The psychological component of endometriosis pain requires further exploration with the aim of building psychological models that can underpin targeted interventions for distress and social withdrawal.

### Supplementary Information

**Additional file 1.**

**Additional file 2.**
