# Supplementary material for: A thematic synthesis of qualitative studies and surveys of the psychological experience of painful endometriosis
Source: BMC Womens Health. 2024 Jan 18;24:50. doi: 10.1186/s12905-023-02874-3 (PMC10795225; doi:10.1186/s12905-023-02874-3)
Supplement: Supplementary file 1 — Additional file 1. [file 12905_2023_2874_MOESM1_ESM.docx]

**Additional files**

**1. Search terms**

1. endometriosis.ab,ti.

2. "pelvi* pain".ab,ti.

3. endometriosis.mp. (or exp Endometriosis/)

4. 1 or 2 or 3

5. pain.mp. or exp Pain/

6. quality of life.ab,ti.

7. experience.ab,ti.

8. qualitative.ab,ti.

9. 6 or 7 or 8

10. 4 and 5 and 9

This exact search strategy was used for Medline, Embase and PsycInfo, PsycExtra and adapted as necessary for ProQuest Dissertations & Theses Global and LILACS.

**2. ENTREQ statement**

| **No Item** | **Guide and description** |  |
| --- | --- | --- |
| 1 Aim | State the research question the synthesis addresses. | P6 End of introduction |
| 2 Synthesis methodol-ogy | Identify the synthesis methodology or theoretical framework which underpins the synthesis, and describe the rationale for choice of methodology (e.g. meta-ethnography, thematic synthesis, critical interpretive synthesis, grounded theory synthesis, realist synthesis, meta-aggregation, meta-study, framework synthesis). | P8-9: Qualitative data synthesis |
| 3 Approach to  searching | Indicate whether the search was pre-planned (comprehensive search strategies to seek all available studies) or iterative (to seek all available concepts until they theoretical saturation is achieved). | P7: Registered on Prospero: see Methods |
| 4 Inclusion criteria | Specify the inclusion/exclusion criteria (e.g. in terms of population, language, year limits, type of publication, study type). | P7: in Methods: |
| 5 Data sources | Describe the information sources used (e.g. electronic databases (MEDLINE, EMBASE, CINAHL, psycINFO, Econlit), grey literature databases (digital thesis, policy reports), relevant organisational websites, experts, information specialists, generic web searches (Google Scholar) hand searching, reference lists) and when the searches conducted; provide the rationale for using the data sources. | P7: in Methods: Search strategy |
| 6 Electronic Search strategy | Describe the literature search (e.g. provide electronic search strategies with population terms, clinical or health topic terms, experiential or social phenomena related terms, filters for qualitative research, and search limits). | P7: in Methods: Search strategy and P32: Additional files Table 1 |
| 7 Study screening  methods | Describe the process of study screening and sifting (e.g. title, abstract and full text review, number of independent reviewers who screened studies). | P7: in Methods: Study selection |
| 8 Study character-istics | Present the characteristics of the included studies (e.g. year of publication, country, population, number of participants, data collection, methodology, analysis, research questions). | P11-12 Results: Qualitative studies, and  P30-31: Table 1 |
| 9 Study selection  results | Identify the number of studies screened and provide reasons for study exclusion (e,g, for comprehensive searching, provide numbers of studies screened and reasons for exclusion indicated in a figure/flowchart; for iterative searching describe reasons for study exclusion and inclusion based on modifications of the research question and/or contribution to theory development). | P11-12 Results: Qualitative studies, and p8 Figure 1. |
| 10 Rationale for  appraisal | Describe the rationale and approach used to appraise the included studies or selected findings (e.g. assessment of conduct (validity and robustness), assessment of reporting (transparency), assessment of content and utility of the findings). | P8 Methods: Quality assessment, and p35 Additional files |
| 11 Appraisal items | State the tools, frameworks and criteria used to appraise the studies or selected findings (e.g. Existing tools: CASP, QARI, COREQ, Mays and Pope [25]; reviewer developed tools; describe the domains assessed: research team, study design, data analysis and interpretations, reporting). | P8 Results: Quality assessment describes COREQ & CASP combination, shown on p35 Additional files Table 3 |
| 12 Appraisal process | Indicate whether the appraisal was conducted independently by more than one reviewer and if consensus was required. | P8: Methods: Quality assessment |
| 13 Appraisal results | Present results of the quality assessment and indicate which articles, if any, were weighted/excluded based on the assessment and give the rationale. | P30-31 for Results and p36-7 Additional files.  P12 Methods: Quality assessment states that no weighting or exclusion based on assessment |
| 14 Data extraction | Indicate which sections of the primary studies were analysed and how were the data extracted from the primary studies? | P8-9: Methods: Qualitative data synthesis |
| 15 Software | State the computer software used, if any. | P9: Methods: Qualitative data synthesis |
| 16 Number of reviews | Identify who was involved in coding and analysis. | P9 Methods: Qualitative data synthesis |
| 17 Coding | Describe the process for coding of data (e.g. line by line coding to search for concepts). | P9 Methods: Qualitative data synthesis |
| 18 Study comparison | Describe how were comparisons made within and across studies (e.g. subsequent studies were coded into pre-existing concepts, and new concepts were created when deemed necessary). | P9 Methods: Qualitative data synthesis |
| 19 Derivation of themes | Explain whether the process of deriving the themes or constructs was inductive or deductive. | P9 Methods: qualitative data synthesis |
| 20 Quotations | Provide quotations from the primary studies to illustrate themes/constructs, and identify whether the quotations were participant quotations of the author’s interpretation. | P13-16 Results |
| 21 Synthesis output | Present rich, compelling and useful results that go beyond a summary of the primary studies (e.g. new interpretation, models of evidence, conceptual models, analytical framework, development of a new theory or construct). | P16-20 Discussion |

**3. Combined CASP/COREQ rating form**

| **Domain/Item** | **Guide questions/description** |
| --- | --- |
| Interviewer/facilitator | Which author/s conducted the interview or focus group? |
| Gender | Was the researcher male or female? |
| Experience and training | What experience or training did the researcher have? |
| Was relationship between researchers & participants adequately considered? (CASP) | Consider • If the researcher critically examined their own role, potential bias and influence during (a) formulation of the research questions (b) data collection, including sample recruitment and choice of location • How the researcher responded to events during the study and whether they considered the implications of any changes in the research design |
| Interviewer characteristics | What characteristics were reported about the interviewer/ facilitator? e.g. Bias, assumptions, reasons and interests in the research topic |
| Methodological orientation and theory | What methodological orientation was stated to underpin the study? e.g. grounded theory, discourse analysis, ethnography, phenomenology, content analysis |
| Clear statement of research aims? (CASP) | Consider • what was the goal of the research • why it was thought important • its relevance |
| Is qual methodol appropriate? (CASP) | Consider • If the research seeks to interpret or illuminate the actions and/or subjective experiences of research participants • Is qualitative research the right methodology for addressing the research goal |
| Sampling | How were participants selected? e.g. purposive, convenience, consecutive, snowball and was recruitment strategy appropriate to research aims (CASP) |
| Sample size | How many participants were in the study? |
| Description of sample | What are the important characteristics of the sample? e.g. demographic data, date |
| Interview guide | Were questions, prompts, guides provided by the authors? Was it pilot tested? |
| Was data analysis sufficiently rigorous? (CASP) | Consider • If there is an in-depth description of the analysis process • If thematic analysis is used. If so, is it clear how the categories/themes were derived from the data • Whether the researcher explains how the data presented were selected from the original sample to demonstrate the analysis process • If sufficient data are presented to support the findings • To what extent contradictory data are taken into account • Whether the researcher critically examined their own role, potential bias and influence during analysis and selection of data for presentation |
| Number of data coders | How many data coders coded the data? |
| Derivation of themes | Were themes identified in advance or derived from the data? |
| Clear statement of findings? (CASP) | Consider whether • If the findings are explicit • If there is adequate discussion of the evidence both for and against the researcher’s arguments • If the researcher has discussed the credibility of their findings (e.g. triangulation, respondent validation, more than one analyst) • If the findings are discussed in relation to the original research question |
| How valuable is res? (CASP) | Consider • If the researcher discusses the contribution the study makes to existing knowledge or understanding (e.g. do they consider the findings in relation to current practice or policy, or relevant researchbased literature • If they identify new areas where research is necessary • If the researchers have discussed whether or how the findings can be transferred to other populations or considered other ways the research may be used |

**4 Table of CASP/COREQ ratings of included studies**

| **Author** | **Title** | **Year** | **Theory; method** | **Interviewer/Facilitator (Gender)** | **Reflexivity** | **Number of data coders** |
| --- | --- | --- | --- | --- | --- | --- |
| Bento & Moreira | Quando os olhos não veem o que as mulheres sentem: a dor nas narrativas de mulheres com endometriose | 2018 | Narratives of life (Bertaux, 2010); not stated | Unclear – most likely Moreira (F) | Limited but present | 2 |
| Boersen et al. | Patients’ perspective on cognitive behavioural therapy after surgical treatment of endometriosis: a qualitative study | 2021 | Not stated; qualitative | First author Boersen ran focus groups; another took notes (M) | None | 2 coded then discussed with 3rd. |
| Bullo & hearne | Parallel worlds and personified pain: A mixed methods analysis of pain metaphor use by women with endometriosis | 2021 | Linguistics; Interpretative Phenomenological Analysis combined with Conceptual Metaphor Theory | Stella Bullo – one-on-one online interviews (F) | Limited but present | 1 |
| Clark | Experiences of women with endometriosis: An Interpretative Phenomenological Analysis. | 2012 | Not stated; qualitative, IPA | Sole author (dissertation) (F) | Good | 1 |
| Cole et al. | “The most lonely condition I can imagine”: Psychosocial impacts of endometriosis on women’s identity | 2020 | Biographical disruption, Feminist, constructions of identity; thematic analysis | n/a online qualitative survey – lead author (F) | Limited but present | 3 |
| Cox et al. | Focus group study of endometriosis:  Struggle, loss and the medical merry-go-round | 2003 | Not stated; qualitative | Focus groups run by first author Cox, with steering group member also in group (F) | None | Not clear: one or two |
| Denny | Women’s experience of endometriosis | 2004 | Not stated; qualitative - thematic and content analysis | Sole author (F) | None | 1 |
| Di biasi | The meaning of endometriosis to females experiencing the disease | 1995 | Hermeneutics, 'demythologising'; not stated | Sole author (F) | Good | 2: author and supervisor. Also help from endometriosis support group |
| Dibeneddetti | Patients’ perspectives of endometriosis related fatigue: qualitative interviews | 2020 | Not stated; Concept elicitation methodology | Two of the authors – DD and CG (F) | Good | 3 – 1 coded all, then 2 coded 10% of transcripts |
| Drabble et al. | Constellations of pain: a qualitative study of the complexity of women’s endometriosis-related pain | 2021 | Not stated; qualitative | Author who was MSc student under supervision of first author did 10, qualitative researcher did 10 (F) | None | Thematic analysis by pairs for first and second 10; one of pair same for all 20. |
| Eastwood | Endometriosis: Medical Delegitimation and the Reconstruction of Narrative Identity | 2005 | Narrative, non-reductionist, feminist, grounded theory; not stated | Sole author who was PhD student (F) | Good | Sole author only: no mention of involving anyone else |
| Gater et al. | Development and content validation of two new patient-reported outcome measures for endometriosis: the Endometriosis Symptom Diary (ESD) and Endometriosis Impact Scale (EIS) | 2020 | Not stated; Thematic Analysis | Experienced interviewers (F) | None | Two per language (2x3)- 6 |
| Guan et al | The endometriosis daily diary: qualitative  research to explore the patient experience of endometriosis and inform the development of a patient-reported outcome (PRO) for endometriosis-related pain | 2022 | Not stated; Thematic Analysis | Not stated (F) | Limited but present | Unclear – possibly all 8 researchers |
| Hallstam | Living with painful endometriosis – A struggle for coherence. A qualitative study | 2018 | Not stated; qualitative, Grounded Theory | Hallstam (15) and Lofgren (1) (F) | Good | 2 |
| Hudson | Endometriosis: improving the wellbeing of couples | 2013 | Not stated | Not stated (n/a) | None | Unclear |
| Huntingdon & Gilmour | A life shaped by pain: women and endometriosis | 2005 | General feminist research principles; Thematic Analysis | Huntingdon (F) | Good | Unclear |
| Jaeger et al | “A little monster inside me that comes out now and again”: endometriosis and pain in Austria | 2022 | Not stated; Qualitative content analysis | Ines Fleischander (n/a) | None | Unclear |
| Jones et al | The impact of endometriosis upon quality of life: a qualitative analysis | 2004 | Not stated; Grounded theory | Not stated (n/a) | Limited but present | Unclear |
| Manderson et al | Circuit Breaking: Pathways of Treatment Seeking for Women With Endometriosis in Australia | 2008 | Belief systems, theories on chronic illness, ideas around the female "normal" body; Grounded Theory | Not stated (n/a) | None | 3 |
| Marki et al | Challenges of and possible solutions for living with endometriosis: a qualitative study | 2022 | Post positivist qualitative paradigm; IPA | Marki (and trained assistant, who took field notes) (F) | None | 2 |
| Markovic et al. | Endurance and contest: women’s narratives of endometriosis | 2008 | Not stated; Grounded Theory | Not stated (n/a) | None | Unclear |
| Matias-Gonzales et al | “Es que tú eres una changa”: stigma experience | 2021 | Not stated; Thematic Analysis | Matias-gonzalez and sanchez-galarza (n/a) | None | 4 |
| Mellado et al | Social isolation in women with endometriosis and chronic pelvic pain | 2015 | Sociology; Grounded Theory | 3 staff per focus group – Mellado or Candido-dos reis chair or taking notes, and Falcone recording (2F, 1M) | None | Unclear |
| Moradi | Impact of endometriosis on women’s lives: a qualitative study | 2014 | Not stated; Thematic Analysis | Focus groups conducted by healthcare professionals, two researchers present taking notes – but also ‘MM organized and guided the focus groups and conducted the initial data analysis. MP attended focus group discussions as the second facilitator’ (n/a) | None | 5 |
| Olliges et al. | The Physical, Psychological, and Social Day-to-Day Experience of Women Living With Endometriosis Compared to Healthy Age-Matched Controls—A Mixed-Methods Study | 2021 | Not stated; IPA | Not stated (n/a) | None | 2 |
| Osborne | The effects of symptomatic endometriosis on womanhood | 2008 | Not stated; IPA | Osborne (F) | Good | 1 |
| Rea et al. | Living with endometriosis: a phenomenological study | 2020 | Phenomenological; not stated | Unclear – 2 of the researchers (n/a) | Good | Unclear |
| Riazi et al. | Patients’ and physicians’ descriptions of occurrence and diagnosis of endo-metriosis: a qualitative study from Iran | 2014 | Cultural context of disease; Content analysis, thematic approach | Riazi (F) | None | 1, but supervised by another researcher |
| Rowe et al. | Improving clinical care for women with endometriosis: qualitative analysis of women’s and health professionals’ views | 2021 | Framework analysis technique; not stated | Rowe (F) | None | Unclear |
| Silva et al | Experiences of women regarding their pathways to the diagnosis of endo-metriosis | 2021 | Thematic/category content research; qualitative | Carla Marins Silva, Camilla  Freitas da Cunha, Karoline Rangel Neves (F) | None | 2 |
| Varney | Women’s experiences of endometriosis: Qualitative explorations of psychological support, and interactions with healthcare professionals | 2020 | Critical realist stance; Inductive reflexive thematic analysis – Braun & Clark | Varney (F) | Good | 1 (but discussed with others) |
| Zale et al | Shedding light on endometriosis: Patient and provider perspectives on a challenging disease | 2019 | Not stated; Thematic Analysis | Not stated (n/a) | None | 2 |
| Zarbo | Cognitive and Personality Factors Implicated in Pain Experience in Women With Endometriosis: A mixed method study | 2019 | Biopsychosocial model; IPA | Unclear – not an author from group (n/a) | None | Unclear – researchers – of which there are 13 named |

**Surveys not referenced in text**

Armour M, Middleton A, Lim S, Sinclair J, Varjabedian D & Smith CA. Dietary Practices of Women with Endometriosis: A Cross-Sectional Survey. The Journal of Alternative and Complementary Medicine. 2021;27:9:771-777.

Ferreira-Valente A, Garcia IQ, Rosa AM, Pereira A, Pais-Ribeiro JL & Jensen MP. The Portuguese 35-item Survey of Pain Attitudes applied to Portuguese women with Endometriosis. Scand J Pain. 2019;19:3:553-563.

Gupta J, Cardoso L, Kanselaar S, Scolese AM, Hamiddadin A, Pollack AZ & Earnshaw VA. Life Disruptions, Symptoms Suggestive of Endometriosis, and Anticipated Stigma Among College Students in the United States. Women’s Health Reports: 2021;2:1:633-642.

Mundo-López A et al. Contribution of Chronic Fatigue to Psychosocial Status and Quality of Life in Spanish Women Diagnosed with Endometriosis. International Journal of Environmental Research and Public Health. 2020;17:11:3831.

O’Hara R, Rowe H & Fisher J. Self-management factors associated with quality of life among women with endometriosis: a cross-sectional Australian survey. Human Reproduction. 2021;36:3:647-655.
